# Electrophysiological Changes in Depressive Patients with Non-Suicidal Self-Injury: An Event-Related Potential Study and Source Analysis

**DOI:** 10.1192/bjo.2025.10227

**Published:** 2025-06-20

**Authors:** Sehoon Shim

**Affiliations:** Soonchunhyang University Cheonan Hospital, Cheonan, Korea, Republic of

## Abstract

**Aims:** Non-suicidal self-injury (NSSI) has been increasingly observed among adolescents as a maladaptive coping mechanism to alleviate emotional distress. Despite its high prevalence, the neurobiological underpinnings linking interpersonal distress to cognitive control deficits remain underexplored. Electroencephalography (EEG) studies suggest that the no-go P3 component may serve as a biomarker for impulsivity and response inhibition, offering insights into the mechanisms underlying NSSI behaviours. This study aimed to investigate the relationship between psychological characteristics, neural activity, and cognitive control in adolescents with NSSI compared with healthy controls (HC).

**Methods:** A total of 51 adolescents with NSSI and 50 HC were recruited. Psychological characteristics were assessed using standardized scales, including the Interpersonal Needs Questionnaire (INQ) and Short UPPS-P Impulsivity Scale (SUPPS-P). EEG was recorded during a go/no-go task to measure P3 amplitudes. Source analysis was performed to localize neural activity. Group differences were analyzed using ANCOVA to control depression and anxiety, followed by partial correlation and mediation analyses to evaluate relationships among variables.

**Results:** The NSSI group exhibited significantly lower no-go P3 amplitudes at all electrodes compared with the HC group (p<0.001), even after controlling for depression and anxiety. No-go P3 amplitudes were negatively correlated with INQ scores, suggesting that interpersonal distress impacts response inhibition. Source analysis revealed reduced neural activity in the right superior frontal gyrus, inferior parietal gyrus, and other regions associated with cognitive control and emotional regulation in the NSSI group. However, these differences disappeared after adjusting for depression and anxiety, indicating their potential mediating role.

**Conclusion:** These findings highlight the interplay between interpersonal distress, depression, anxiety, and cognitive control deficits in adolescents with NSSI. Future longitudinal studies are needed to confirm these pathways and explore therapeutic interventions targeting interpersonal distress and emotional regulation to mitigate NSSI behaviours.

